# Amyloid Positron Emission Tomography Imaging at the Onset of Amyloid-Related Abnormalities With Edema in an Alzheimer’s Disease Patient

**DOI:** 10.7759/cureus.100276

**Published:** 2025-12-28

**Authors:** Heii Arai, Hideo Yamamoto, Yuki Akiba, Satoko Aiba, Reiko Arai

**Affiliations:** 1 Department of Psychiatry, ALZClinic Tokyo, Tokyo, JPN; 2 Department of Radiology, ALZClinic Pet Lab, Tokyo, JPN; 3 Department of Psychiatry, ALZClinic Pet Lab, Tokyo, JPN

**Keywords:** alzheimer’s dementia, amyloid pet, aria-e, donanemab, lecanemab, mri imaging

## Abstract

Amyloid-related imaging abnormalities with edema (ARIA-E) are known adverse events of anti-amyloid monoclonal antibody therapy for Alzheimer’s disease (AD), but to our knowledge, amyloid positron emission tomography (PET) captured exactly at the moment of ARIA-E onset has not previously been reported. We report an 85-year-old woman with AD who received anti-amyloid antibody therapy for approximately one year. As part of routine post-treatment evaluation, a second amyloid PET scan obtained 16 days after her final infusion unexpectedly revealed asymptomatic ARIA-E when followed immediately by magnetic resonance imaging (MRI). Compared with the baseline, the follow-up PET showed a marked overall reduction in cortical amyloid burden, including near-complete loss of tracer uptake in the region corresponding to ARIA-E, while the adjacent white matter exhibited reduced, non-specific uptake likely related to edema. This unique, same-day PET-MRI pairing at the moment of ARIA-E detection highlights dynamic regional changes in amyloid signal during treatment and emphasizes the value of continued neuroimaging surveillance even in asymptomatic patients.

## Introduction

In Japan, the Optimal Use Guideline for Anti-Amyloid Antibody Therapy for donanemab recommends reassessment of amyloid burden with positron emission tomography (PET) approximately one year after treatment initiation to determine whether amyloid has normalized sufficiently to consider treatment completion [1]. This principle aligns with the modern biological framework of Alzheimer’s disease (AD), which emphasizes biomarker-based staging and the dynamic trajectory of amyloid accumulation and clearance [2].

Anti-amyloid monoclonal antibodies, such as lecanemab and donanemab, have demonstrated clinical efficacy in mild cognitive impairment and early AD through the reduction of cerebral amyloid burden [3,4]. Recent therapeutic reviews indicate a class-wide effect of robust amyloid lowering across multiple monoclonal antibodies, such as plaque-targeting and protofibril-targeting agents, including aducanumab, donanemab, and lecanemab [5]. A known risk of these therapies is the development of amyloid-related imaging abnormalities (ARIA), including ARIA with edema (ARIA-E), reflecting vasogenic edema, and ARIA with hemorrhage (ARIA-H), characterized by microhemorrhages or superficial siderosis. ARIA has been well characterized in clinical trials-particularly those of aducanumab, where detailed analyses clarified radiologic patterns, time courses, and risk factors [6].

ARIA typically manifests within the first few months of treatment. While magnetic resonance imaging (MRI) is routinely used to detect ARIA and prior studies have described its characteristic radiological features [7], the relationship between amyloid dynamics, vascular inflammation, and ARIA pathophysiology remains incompletely understood. In this context, amyloid PET may provide complementary information by capturing regional amyloid burden and its dynamic changes during treatment, which could be relevant for clinical monitoring and decision-making. In particular, recent literature on cerebral amyloid angiopathy (CAA) suggests that inflammatory responses and regional amyloid shifts may contribute to imaging abnormalities [8].

However, no case has previously reported amyloid PET findings obtained contemporaneously with ARIA onset. We present a case of an elderly woman with AD who developed asymptomatic ARIA at the end of sequential lecanemab and donanemab treatment. Notably, amyloid PET demonstrated a marked reduction in cortical amyloid deposition with essentially complete loss of signal in the ARIA-affected region.

## Case presentation

An 85-year-old woman presented with a three-year history of progressive memory impairment and was first evaluated at our clinic in April 2024. On initial examination, her Mini-Mental State Examination (MMSE) [9] score was 20, and her Clinical Dementia Rating-Global Score (CDR-GS) [10] was 1. Brain MRI revealed general cortical atrophy, including the hippocampus, with only mild vascular changes. Amyloid PET using florbetapir® showed cortical amyloid deposition consistent with AD pathology.

Anti-amyloid β monoclonal antibody therapy was initiated in May 2024. The patient was followed concurrently by an internist, and medications including candesartan and rosuvastatin were continued. Aspirin was switched to edoxaban before initiating anti-amyloid therapy. She received 12 infusions of lecanemab through November 2024 and subsequently requested transition to donanemab to reduce infusion time and frequency, with additional consideration given to its monthly dosing schedule and plaque-targeting pharmacological profile, while continuing careful monitoring for ARIA. She then received six monthly donanemab infusions until June 2025. Infusion-related reactions (IRRs) were limited to mild headaches after the first two lecanemab infusions, which resolved with acetaminophen. No IRRs occurred during donanemab treatment.MRI and PET at follow-up were performed 16 days after the final donanemab infusion. During the interval between the final infusion and imaging, the patient was clinically monitored and reported no new neurological symptoms, headaches, focal deficits, or changes in daily functioning. The timing of the second PET scan followed the national Optimal Use Guideline, which recommends repeating amyloid PET approximately one year after treatment initiation to determine whether amyloid burden has normalized enough to discontinue therapy [1]. Amyloid PET was performed using 18F-florbetapir and visually interpreted by two licensed physicians according to established criteria [11] with quantitative analyses including standardized uptake value ratios (SUVR) and Centiloid (CL) conversion [12]. Structural MRI was obtained using a standard 3D T1-weighted sequence, and medial temporal atrophy was assessed using the Voxel-based Specific Regional Analysis System for Detection of Alzheimer’s disease (VSRAD), which quantifies regional gray matter loss relative to an age-matched normative database. Detailed PET acquisition, reconstruction, and MRI analysis protocols have been described in our previous work [11]. 

Clinical and neuroimaging data were retrospectively reviewed after ARIA onset. MRI was evaluated for ARIA-E, and PET quantitative indices (CL and SUVR) were compared with baseline findings. Written informed consent for publication of de-identified clinical data and images was obtained from the patient and her family.

**Figure 1:**
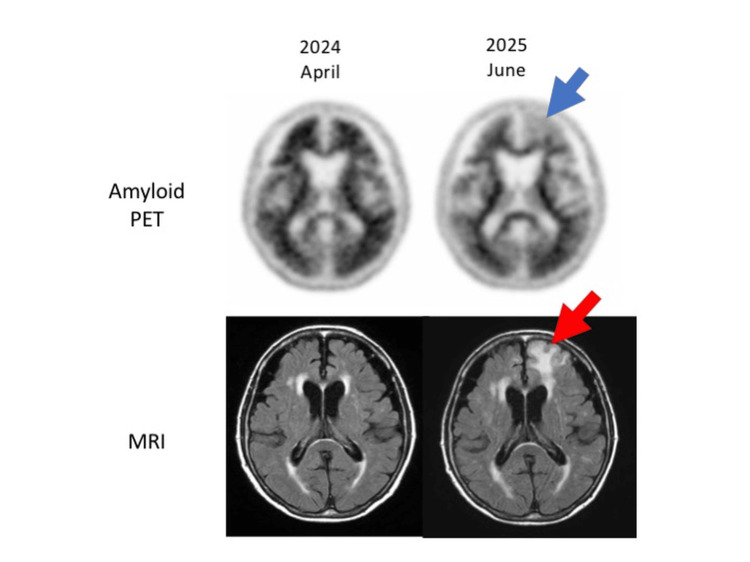
Amyloid PET and FLAIR MRI at baseline and at ARIA-E onset. Serial amyloid PET and MRI obtained at baseline (April 2024) and at ARIA-E onset (June 2025). Upper panels show 18F-florbetapir PET images demonstrating a marked reduction in cortical amyloid burden at ARIA-E onset; the blue arrow indicates the region of near-complete tracer uptake loss in the left frontal cortex, corresponding to the site of ARIA-E. Lower panels show fluid-attenuated inversion recovery (FLAIR) MRI, in which the red arrow highlights a new left frontal hyperintensity consistent with ARIA-E.

Serial MRI scans up to April 2025, performed as part of routine safety monitoring in accordance with the national Optimal Use Guideline for anti-amyloid antibody therapy [1] and applied uniformly to all treated patients, demonstrated no evidence of ARIA. However, at the end of the one-year treatment period, follow-up MRI revealed new ARIA-E lesions predominantly in the frontal lobes (Figure 1).

Importantly, the patient remained clinically asymptomatic, without changes in physical or neuropsychological status. In accordance with the scheduled one-year evaluation, amyloid PET was performed first, followed immediately by MRI on the same day. Importantly, because the PET scan was completed prior to MRI, the newly detected ARIA-E on MRI did not influence the decision to perform PET. Compared to baseline, follow-up amyloid PET showed a substantial reduction in cortical amyloid burden (Figure 1), with decreased Centiloid values and reductions across all SUVR-derived regions of interest. Notably, the ARIA-affected region demonstrated essentially complete loss of cortical amyloid signal, contrasting with the high tracer retention observed at baseline. VSRAD analysis revealed progression of atrophy, with increases in severity score and volume-of-interest (VOI) atrophy (Table 1). Cognitively, the patient’s MMSE declined slightly from 20 to 18, but the CDR-GS remained stable at 1.

**Table 1:** Summary of Amyloid PET and VSRAD Measures at Baseline and ARIA Onset. Amyloid PET and VSRAD summary values at two time points: baseline (April 2024) and at ARIA onset (June 2025). Centiloid and SUVR values reflect amyloid burden in specific brain regions, while VSRAD metrics quantify medial temporal and global gray matter atrophy. Percentage change was calculated as: (value at ARIA onset-baseline value)/baseline value × 100. SUVR: standardized uptake value ratio, VSRAD: Voxel-based specific regional analysis system for detection of Alzheimer's disease, GM: gray matter, VOI: volume of interest.

Date	Centiloid	SUVR Global	SUVR Frontal	SUVR Temporal	SUVR Post. Cingulate	SUVR Parietal	VSRAD Severity	VSRAD GM Atrophy (%)	VSRAD VOI Atrophy (%)	VOI/GM Ratio
Apr 2024 (Baseline)	85.4	1.528	1.436	1.649	1.585	1.639	2.76	5.11	70.44	13.78
Jun 2025 (At ARIA onset)	6.3	1.076	0.965	1.267	1.099	1.194	3.59	7.7	89.29	11.6
% Change from Baseline	−92.6%	−29.6%	−32.8%	−23.2%	−30.7%	−27.1%	30.10%	50.70%	26.80%	−15.8%

## Discussion

To our knowledge, this is the first report to document amyloid PET imaging obtained at the time of ARIA-E detection. Our case demonstrated near-complete loss of cortical amyloid PET signal specifically within the ARIA-affected region, which previously exhibited high tracer uptake. This observation may reflect a regionally accentuated antibody-mediated amyloid clearance mechanism apparent at the end of sequential anti-amyloid therapy. Previous mechanistic studies of monoclonal antibody therapy suggest that rapid mobilization of amyloid may exceed vascular clearance capacity, leading to transient blood-brain barrier dysfunction and vasogenic edema [13].

The patient had no prior history of ARIA and tolerated both lecanemab and donanemab well. The detection of ARIA exclusively on imaging obtained 16 days after the final donanemab infusion suggests a delayed biological effect related to cumulative amyloid clearance. Similar delayed or treatment-phase-dependent ARIA patterns have been reported in aducanumab clinical trials, where ongoing amyloid reduction was associated with the late emergence of ARIA in a subset of patients [14].

The marked decrease in CL values supports a mechanism in which extensive amyloid reduction may contribute to ARIA-E even in asymptomatic patients. Previous reports involving cerebral amyloid angiopathy-related inflammation (CAA-ri) have described focal reductions in amyloid PET uptake within ARIA-E regions, typically without complete loss of signal and often unrelated to areas of highest baseline amyloid burden [15]. Additional CAA-ri case series have further shown that acute vascular inflammation and blood-brain barrier dysfunction can transiently modify regional tracer kinetics, producing apparent decreases that do not necessarily reflect true changes in fibrillar amyloid [16]. These mechanisms provide a plausible explanation for our findings and underscore the importance of interpreting focal PET reductions within the broader context of concurrent MRI abnormalities and inflammatory activity rather than attributing them solely to accelerated therapeutic amyloid removal.

Differences in disease context, imaging timing, or treatment exposure may explain the more pronounced finding observed in our case. Non-specific tracer reduction in adjacent white matter was also observed, likely reflecting ARIA-related edema or altered tracer delivery rather than true changes in amyloid burden. Prior PET-MRI correlation studies have shown that vasogenic edema can reduce apparent tracer uptake through partial-volume effects or impaired perfusion [17].

Collectively, these findings highlight the ability of amyloid PET to capture treatment-related biological dynamics and underscore the importance of understanding microvascular responses during immunotherapy. They also suggest that scheduled imaging surveillance may help identify subclinical ARIA-related changes, even in the absence of overt neurological symptoms. Recent multimodal imaging reviews emphasize that integrating PET with MRI improves characterization of amyloid removal kinetics, vascular inflammation, and ARIA progression [18].

Advanced age is a recognized risk factor for ARIA, and although ARIA is commonly considered an early-phase complication of anti-amyloid therapy, we recently reported a low incidence of ARIA during the first five donanemab infusions in 20 patients who had previously undergone approximately one year of lecanemab therapy [19]. Despite the younger mean age (73 years) in that cohort, ARIA in the present case emerged after six donanemab infusions. Other longitudinal observational studies similarly report that advanced age increases susceptibility to ARIA, particularly during later treatment phases [20].

Further studies are warranted to clarify interactions between amyloid dynamics and ARIA risk across treatment timelines. Emerging research suggests that vascular amyloid burden, microglial activation state, and APOE genotype may modulate ARIA vulnerability. Additional PET studies examining microglial-vascular interactions further highlight potential mechanisms linking immune activation to ARIA occurrence [11].

## Conclusions

This case demonstrates that amyloid PET performed at the moment of ARIA-E can reveal distinct regional tracer loss that is not usually visible on routine follow-up scans. The paired same-day acquisition of positron emission tomography and magnetic resonance imaging highlighted a localized disappearance of cortical signal despite the patient remaining clinically stable. These findings suggest that real-time molecular imaging may capture dynamic biological changes occurring during antibody treatment. Overall, this case underscores the importance of ongoing imaging surveillance during and after anti-amyloid therapy, particularly in older individuals or those receiving sequential treatment.
